# Mental and Behavioural Disorders of Childhood and Adolescence: An Observational Study

**DOI:** 10.9734/jammr/2021/v33i1631015

**Published:** 2021-07-23

**Authors:** Shyamanta Das, Soumitra Ghosh, Dhrubajyoti Bhuyan, Hiranya Saikia, Hiranya Kumar Goswami, Rupi Varsha Soren, Samrat Singh Bhandari

**Affiliations:** 1 Department of Psychiatry, Gauhati Medical College Hospital, Guwahati, Assam, India.; 2 Department of Psychiatry, Tezpur Medical College Hospital, Tezpur, Assam, India.; 3 Department of Psychiatry, Assam Medical College Hospital, Dibrugarh, Assam, India.; 4 Department of Community Medicine, Assam Medical College Hospital, Dibrugarh, Assam, India.; 5 Department of Psychiatry, Sikkim Manipal Institute of Medical Sciences, Gangtok, Sikkim, India.

**Keywords:** Psychiatry, Comorbidity, Age, Sex

## Abstract

**Background:**

There is overlap of symptoms in psychiatric disorders, especially in mental and behavioural disorders of childhood and adolescence. Half of all lifetime psychiatric disorders tend to arise by age 14 years and three fourths of them arise by age 24 years.

**Aim:**

To study the various types of mental and behavioural disorders of childhood and adolescence, and to find out comorbidities within and across the types.

**Methods:**

An observational cross-sectional study was carried out over a period of one year in the psychiatry department of a tertiary care general hospital. The psychiatric diagnoses according to the World Health Organization’s (WHO) tenth revision of the International Statistical Classification of Health and Related Problems (ICD-10) were categorised into type 1 (depression, anxiety, obsessive-compulsive disorder, and somatoform disorder), type 2 (attention-deficit/hyperactivity disorder, oppositional defiant disorder, and conduct disorder), type 3 (mental retardation, developmental disorders of speech and language, and scholastic skills, and pervasive developmental disorders). Descriptive statistics was used with frequency and percentage.

**Results:**

Total sample size was 137. Children and adolescents were almost equally distributed. Boys were more than girls. Type 3 disorders were maximum. Adolescents had mostly type 1 disorders. Children had mostly type 3 disorders. Girls had almost same number of type 1 and type 3 disorders. Boys had mostly type 3 disorders. Within group comorbidity was mostly with type 3 disorders. Across group comorbidity was highest in type2-type 3 disorders.

**Conclusion:**

Mental and behavioural disorders in childhood and adolescence do vary according to age and sex, and their recognition will help in the early diagnosis and proper management.

## 1. INTRODUCTION

Mental and behavioural disorders of childhood and adolescence is a reality or is it a myth? Half of all lifetime psychiatric disorders tend to arise by age 14 years and three fourths of them arise by age 24 years [1]. It is a common observation that there is overlap of symptoms in psychiatric disorders. This is more seen, especially in mental and behavioural disorders of childhood and adolescence. For example, depression, anxiety, obsessive-compulsive disorder (OCD), and somatoform disorder have common features. Attention-deficit/hyperactivity disorder (ADHD), oppositional defiant disorder (ODD), and conduct disorder also have common features. Similarly, mental retardation (MR), developmental disorders of speech and language, and scholastic skills, and pervasive developmental disorders (PDD) have common features. And finally, psychotic illnesses including the schizophrenia spectrum and mood disorders which may be unipolar or bipolar also have similar features. Therefore, in this study, we clubbed the different disorders into type 1 consisting of depression, anxiety, OCD, and somatoform disorder, type 2 consisting of ADHD, ODD, and conduct disorder, type 3 consisting of MR, developmental disorders of speech and language, and scholastic skills, and PDD, and type 4 consisting of psychotic illnesses including the schizophrenia spectrum and mood disorders which may be unipolar or bipolar.

### 1.1 The Objectives of the Present Study Are

1. To study the various types of mental and behavioural disorders of childhood and adolescence.
2. To find out comorbidities within and across the types.

## 2. MATERIALS AND METHODS

### 2.1 Study design

It was an observational cross-sectional study.

### 2.2 Setting

The current study was conducted in Gauhati Medical College Hospital (GMCH), Guwahati, Assam, India. There is a 12-bedded child psychiatry unit (CPU) in GMCH with three beds for each sex in both age groups of up to 12 years and from 12 to 18 years. Also, there is the Child Guidance Clinic (CGC) within the out-patient department (OPD) of psychiatry. CPU and CGC of GMCH were the locations for the present study.

### 2.3 Duration

The study was conducted over a period of one year from 7 September 2018 to 6 September 2019.

### 2.4 Participants

Children and adolescents who were admitted in CPU and who attended CGC with an accompanying guardian giving written informed consent constituted the study participants.

### 2.5 Variables

Both demographic and clinical variables were studied. Demographic variables included age and sex. Clinical variables were the diagnoses according to the World Health Organization’s (WHO) tenth revision of the International Statistical Classification of Diseases and Related Health Problems (ICD-10) [2].

### 2.6 Definition of Outcomes

The ICD-10 [2] diagnoses were categorised into type 1, type 2, type 3, and type 4. Within group and across groups comorbidities were noted. Diagnoses and comorbidities were analysed in relation to the demographic variables of age and sex.

### 2.7 Data Sources/Measurement

#### Sources of data:

Proformas of patients admitted in CPU and attending CGC were the sources of data.

#### Tools:

Tools for the study were the demographic and clinical proformas, as well as ICD-10 [2].

#### Details of methods of assessment (measurement):

Admitted patients in CPU and those attending CGC with their ascent wherever applicable and accompanied by a guardian who gave written informed consent over the study period were assessed and diagnosed according to ICD-10 [2]. Their demographic and clinical data were entered in the respective proformas prepared for the present study. The ICD-10 diagnoses were categorised into type 1, type 2, type 3, and type 4. Within group and across groups comorbidities were noted. Diagnoses and comorbidities were analysed by descriptive statistics in relation to the demographic variables.

### 2.8 Study Size

From the National Mental Health Survey of India, [3] prevalence of mental health disorders in childhood and adolescence is 7.3%. So, considering this with five per cent absolute error (using formula = (Z_(1-α)/2_)^2^P(1-P)/d^2^), the calculated sample size was 104. We have 104 as the minimum sample size but keeping the upper limit open till the study period.

### 2.9 Statistical Methods

Data were analysed by descriptive statistics in the form of frequency and percentage.

## 3. RESULTS

Total sample size was 137.

**Fig. 1.**
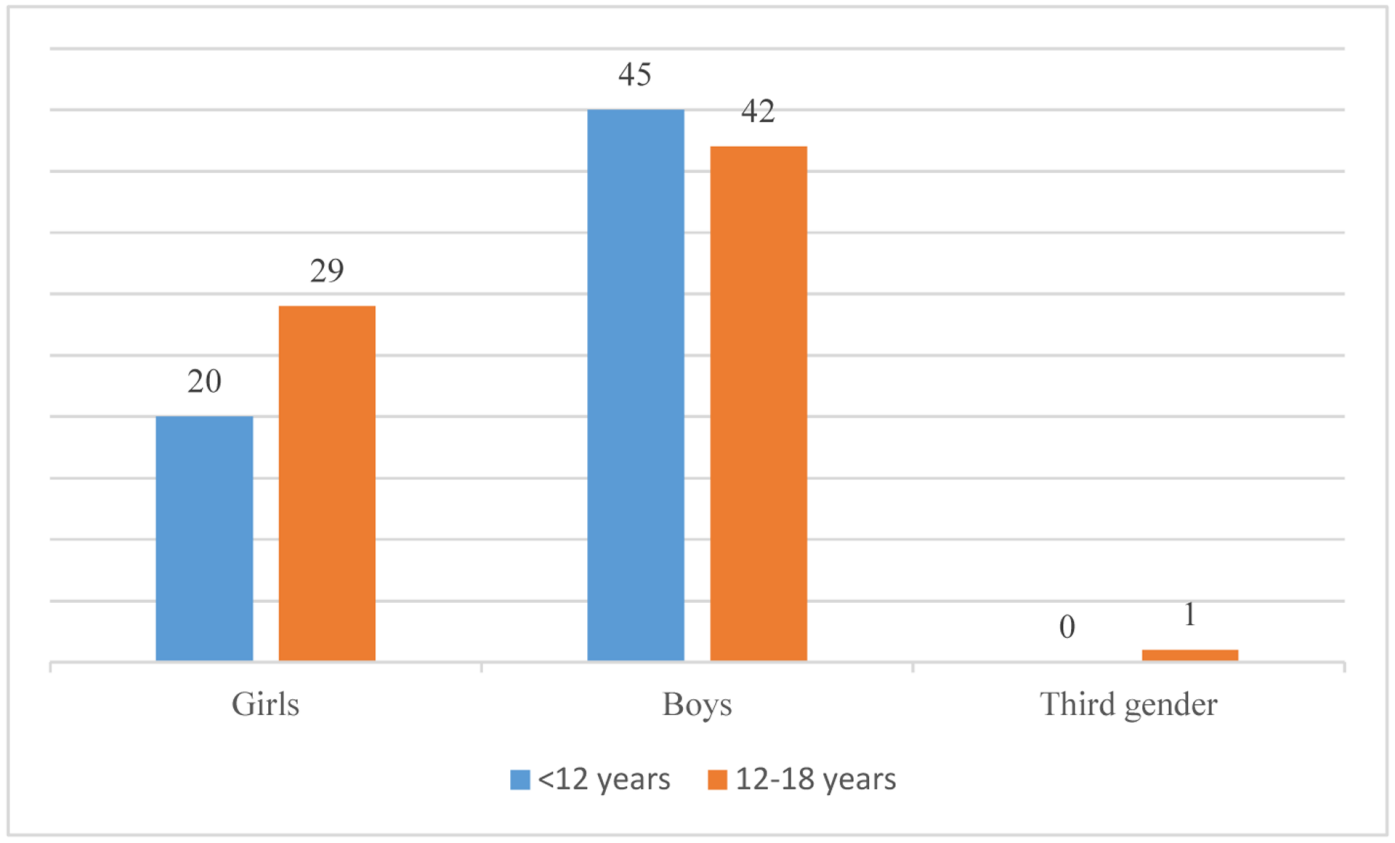
Age and sex distribution of children and adolescents with mental and behavioural disorders

Girls were more below 12 years while boys weremore in the 12 to 18 years age group (Fig. 1).

**Fig. 2.**
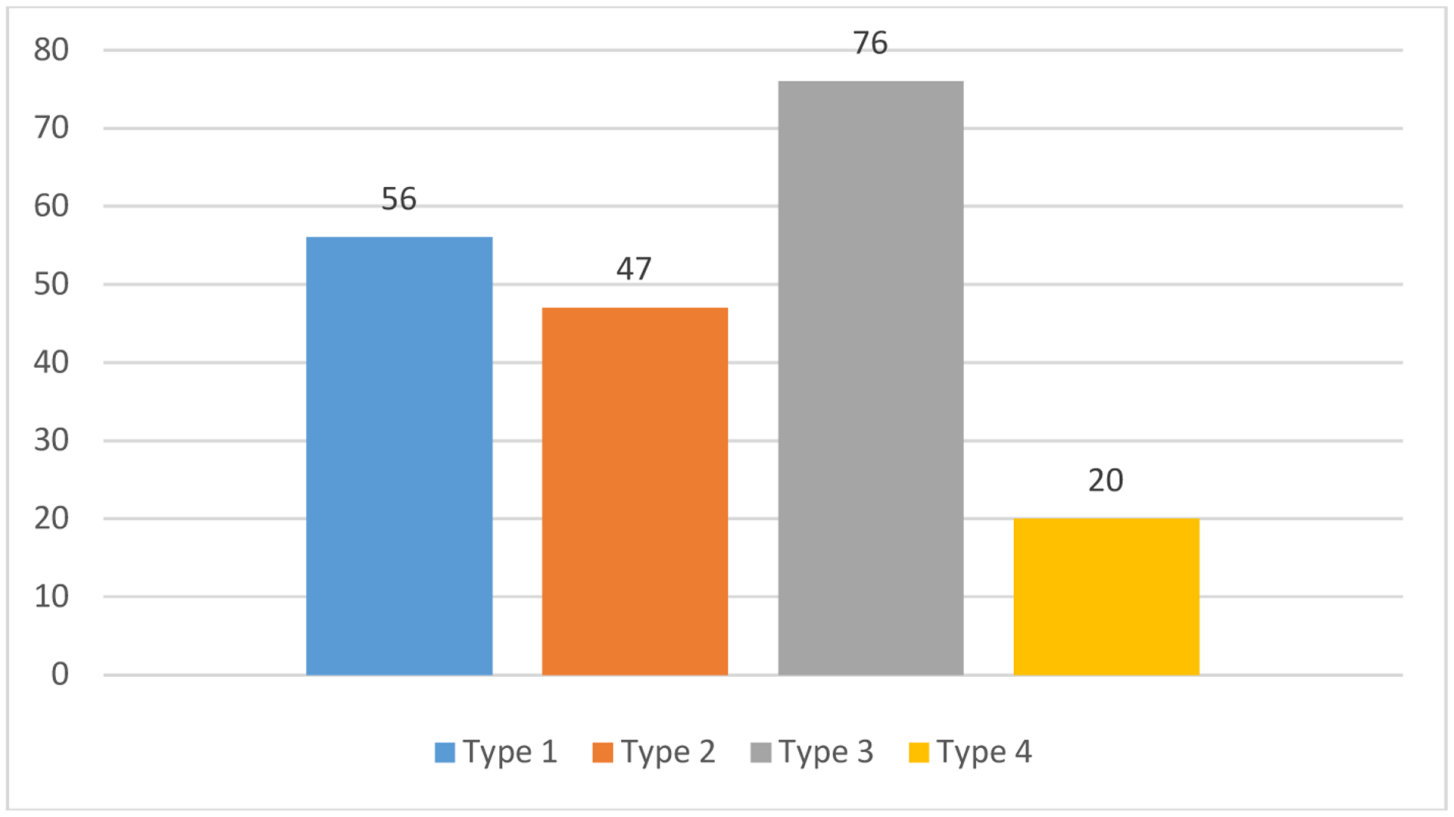
Clinical profile. Number of diagnosis exceeds number of sample as the children and adolescents received more than one diagnosis Type 1: Depression, anxiety, obsessive-compulsive disorder, and somatoform disorder; Type 2: Attention-deficit/hyperactivity disorder, oppositional defiant disorder, and conduct disorder; Type 3: Mental retardation, developmental disorders of speech and language, and scholastic skills, and pervasive developmental disorders; Type 4: schizophrenia spectrum disorders and mood disorders

Most of the children and adolescent were having type 3 disorders, followed by type 1 and type 2 disorders (Fig. 2).

While majority had type 3 disorders below 12 years, type 1 disorders were highest in the 12 to 18 years age group (Table 2).

Type 1 and type 3 disorders were almost equally distributed among girls, while boys predominantly had type 3 disorders (Table 3).

Within group comorbidity was maximum with type 3 disorders (Table 4).

Across group comorbidity was found mostly in type 2-type 3 disorders (Table 5).

## 4. DISCUSSION

We studied 137 children and adolescents having mental and behavioural disorders attending the psychiatry department of a tertiary care general hospital over a period of one year.

### 4.1 Age and Sex

In child psychiatry, developmental psychopathology contributed epidemiological data like age and sex as typical validating criteria. ‘Childhood psychosis’ was earlier considered a single entity. Evidence regarding age led to autism and schizophrenia being validated as two separate and distinct disorders. Similarly, sex determined the distinction between disruptive disorders and emotional disorders. Disruptive disorders are common in boys and emotional disorders in girls [4].

We found in girls, adolescents have more mental and behavioural disorders (<12 years : 12–18 years :: 20 : 29) while in boys, children have more mental and behavioural disorders (<12 years : 12–18 years :: 45 : 42).

In children, type 3 disorders were more and in adolescents, type 1 and type 4 disorders were more while type 2 disorders were equally distributed.

The United Kingdom national prevalence study found that almost one in ten children aged five to 15 years had psychiatric disorders based on ICD-10. Prevalence was higher in adolescents (11.2% at 11–15 years) than in children (8.2% at five to ten years) and in boys (11.4%) than girls (7.6%) [5,6]. Other studies from around world usually generate prevalence rates of around 20% [7].

Internet use increased during the Coronavirus disease 2019 (COVID-19) epidemic. Dong *et al* [8]. enrolled 2050 participants with mean age of 12.34±4.67 years. 2.68% participants had addictive Internet use and 33.37% had problematic Internet use. Girl sex, age, depression, and stress were risks for internet addiction.

Colizzi *et al* [9]. collected data on 114 children aged five to 15 years. Eighty three of them had neurodevelopmental disorders (NDD) and 31 were healthy controls. Boys had higher risk for NDD. Moreover, increasing level of intelligence protected from NDD.

### 4.2 Comorbidity

Within group comorbidity is commonplace compared to that of across group [4]. We found 30 within group comorbidity and 24 across groups comorbidity. But, there were 15 children with more than two comorbidities.

## 5. CONCLUSION

Current classificatory systems, ICD-10 and the American Psychiatric Association’s (APA) fifth edition of the Diagnostic and Statistical Manual of Mental Disorders (DSM-5) [10]. Contain few emotional disorders for children and adolescents that are only anxiety-related. The same diagnostic criteria applied for adults for mood disorders is applied for children also. Mental and behavioural disorders in childhood and adolescence do vary according to age and sex, and their recognition will help in the early diagnosis and proper management. This will even help in keeping an eye for new approaches in childhood and adolescence mental and behavioural disorders.

## LIMITATIONS

The sample was collected in a tertiary care hospital and thus, the findings cannot be generalised to the entire population.

## Figures and Tables

**Table 1.** Demography

Age (in years)	
<12	65
12–18	72
Sex	
Girls	49
Boys	87
Third gender	1

Age-wise, the sample was almost equally distributed between those below 12 years and those in 12 to 18 years age group (Table 1). Boys outnumbered girls.

**Table 2.** Childhood and adolescence mental and behavioural disorders in relation to age

Age (in years)	Type 1 disorders	Type 2 disorders	Type 3 disorders	Type 4 disorders
<12	14	24	59	5
12–18	42	23	17	15

Number of diagnosis exceeds number of sample as the children and adolescents received more than one diagnosis; Type 1: Depression, anxiety, obsessive-compulsive disorder, and somatoform disorder; Type 2: Attention-deficit/hyperactivity disorder, oppositional defiant disorder, and conduct disorder; Type 3: Mental retardation, developmental disorders of speech and language, and scholastic skills, and pervasive developmental disorders; Type 4: schizophrenia spectrum disorders and mood disorders

**Table 3.** Sex and diagnosis

Sex	Type 1 disorders	Type 2 disorders	Type 3 disorders	Type 4 disorders
Girls	24	11	26	10
Boys	31	36	50	10
Third gender	1	0	0	0

Number of diagnosis exceeds number of sample as the children and adolescents received more than one diagnosis; Type 1: Depression, anxiety, obsessive-compulsive disorder, and somatoform disorder; Type 2: Attention-deficit/hyperactivity disorder, oppositional defiant disorder, and conduct disorder; Type 3: Mental retardation, developmental disorders of speech and language, and scholastic skills, and pervasive developmental disorders; Type 4: schizophrenia spectrum disorders and mood disorders

**Table 4.** Comorbidity within group

Type 1-type 1 disorders	Type 2-type 2 disorders	Type 3-type 3 disorders	Type 4-type 4 disorders
5	8*	17	0

* One adolescent girl had three disorders; Type 1: Depression, anxiety, obsessive-compulsive disorder, and somatoform disorder; Type 2: Attention-deficit/hyperactivity disorder, oppositional defiant disorder, and conduct disorder; Type 3: Mental retardation, developmental disorders of speech and language, and scholastic skills, and pervasive developmental disorders; Type 4: schizophrenia spectrum disorders and mood disorders

**Table 5.** **Comorbidity across groups** *

Type 1-type 2 disorders	Type 1-type 3 disorders	Type 1-type 4 disorders	Type 2-type 3 disorders	Type 2-type 4 disorders	Type 3-type 4 disorders
3	4	4	10	1	2

* Fifteen children and adolescents had more than two ‘across groups’ comorbidities (13 had three and two had four diagnoses); Type 1: Depression, anxiety, obsessive-compulsive disorder, and somatoform disorder; Type 2: Attention-deficit/hyperactivity disorder, oppositional defiant disorder, and conduct disorder; Type 3: Mental retardation, developmental disorders of speech and language, and scholastic skills, and pervasive developmental disorders; Type 4: schizophrenia spectrum disorders and mood disorders
